# The study of human Y chromosome variation through ancient DNA

**DOI:** 10.1007/s00439-017-1773-z

**Published:** 2017-03-04

**Authors:** Toomas Kivisild

**Affiliations:** 1 Department of Archaeology and Anthropology, University of Cambridge, Cambridge, CB2 1QH UK; 2 Estonian Biocentre, 51010 Tartu, Estonia

## Abstract

High throughput sequencing methods have completely transformed the study of human Y chromosome variation by offering a genome-scale view on genetic variation retrieved from ancient human remains in context of a growing number of high coverage whole Y chromosome sequence data from living populations from across the world. The ancient Y chromosome sequences are providing us the first exciting glimpses into the past variation of male-specific compartment of the genome and the opportunity to evaluate models based on previously made inferences from patterns of genetic variation in living populations. Analyses of the ancient Y chromosome sequences are challenging not only because of issues generally related to ancient DNA work, such as DNA damage-induced mutations and low content of endogenous DNA in most human remains, but also because of specific properties of the Y chromosome, such as its highly repetitive nature and high homology with the X chromosome. Shotgun sequencing of uniquely mapping regions of the Y chromosomes to sufficiently high coverage is still challenging and costly in poorly preserved samples. To increase the coverage of specific target SNPs capture-based methods have been developed and used in recent years to generate Y chromosome sequence data from hundreds of prehistoric skeletal remains. Besides the prospects of testing directly as how much genetic change in a given time period has accompanied changes in material culture the sequencing of ancient Y chromosomes allows us also to better understand the rate at which mutations accumulate and get fixed over time. This review considers genome-scale evidence on ancient Y chromosome diversity that has recently started to accumulate in geographic areas favourable to DNA preservation. More specifically the review focuses on examples of regional continuity and change of the Y chromosome haplogroups in North Eurasia and in the New World.

## Background

Until recently, ancient DNA studies of human remain focused primarily on variation embedded in mitochondrial DNA (mtDNA). For decades, mtDNA had been a target of choice in population genetic studies because of its high mutation rate and high density of polymorphic markers (Wilson et al. 1985). Also, because for any unique sequence in the autosomal, X or Y chromosome locus there are many hundreds or even thousands of copies of mtDNA means that this maternally inherited locus was more likely to work in cases where only a very small number of molecules had survived (Krings et al. 1997). HTS technologies have significantly increased the rate at which sequence data can be generated and make accessible surviving chunks of DNA that are shorter than the size of a PCR (polymerase chain reaction) amplicon (Orlando et al. 2015). They have reduced the costs of sequencing and opened the prospects of assessing the variation of human populations, both modern and ancient, at the scale of entire genomes (Genomes Project et al. 2012; Gilbert et al. 2008; Green et al. 2010; Rasmussen et al. 2010). These technological advances (Orlando et al. 2015) have also made it possible now to study ancient Y chromosome (aY) variation in human populations at the scale of the entire accessible length of the male-specific and non-recombining regions of human Y chromosome.

Ancient DNA presents us the opportunity to directly examine which Y chromosome single nucleotide polymorphisms (SNPs) and haplotypes were present at different time periods in regions that support long-term survival of ancient DNA. It is perhaps not surprising that archaeological sites from high latitude areas (Hofreiter et al. 2015) have yielded the largest number of successfully sequenced samples in recent genome-scale studies that have reported on aY. These studies, as reviewed in further detail below, have provided us the first glimpses of the dynamics of aY haplogroup composition and frequency changes in transects of time and allow us to test hypotheses based on earlier phylogeographic inferences made from the Y chromosome data of presently living populations. In this review, ‘haplogroups’ and ‘clades’ are terms that are interchangeably used to refer to groups of closely-related Y chromosome sequences that share a common ancestor. While there is a general agreement in the definition of the basic haplogroups (A, B, C, etc.) multiple parallel nomenclatures are in use for sub-clades (e.g. see http://www.phylotree.org/Y/, http://isogg.org/tree/, https://www.yfull.com/tree/). For clarity, the names of sub-clades used here are suffixed by the defining SNP-marker name, as suggested previously (Karmin et al. 2015; van Oven et al. 2014). Similarly to the extent to which our earlier views on peopling of continental regions such as Europe based on inferences made from extant mtDNA variation have changed in the light of new ancient mtDNA evidence (Bollongino et al. 2013; Bramanti et al. 2009; Brandt et al. 2013; Haak et al. 2015; Posth et al. 2016; Thomas et al. 2013), it can be seen that models linking the spread of specific Y chromosome haplogroups with the spread of material culture may require substantial revision in the light of new aY evidence.

In this review, methodological aspects of ancient Y chromosome work will be first discussed with a focus on recent HTS research based on shotgun and capture approaches. Challenges common to all ancient DNA studies include those related to calling human polymorphic variants from short and damaged sequence reads that are derived from a mix of different organisms. Highly repetitive nature of the Y chromosome combined with its high sequence homology with the X chromosome (Skaletsky et al. 2003) further complicates variant calling from short ancient DNA sequence reads. These issues, together with the paternal inheritance of Y chromosome, its haploid nature and high linkage between physically distant SNPs, impact on choices of the bioinformatics methods of downstream data analysis. They define the restrictions and specifics how the aY sequence data should be processed and what are the limitations for the interpretations made from such data. Finally, the demographic histories European and North American populations will be briefly reviewed in the light of recently emerged Y chromosome evidence from ancient DNA studies.

## Methodological issues in dealing with aY

**Fig. 1:**
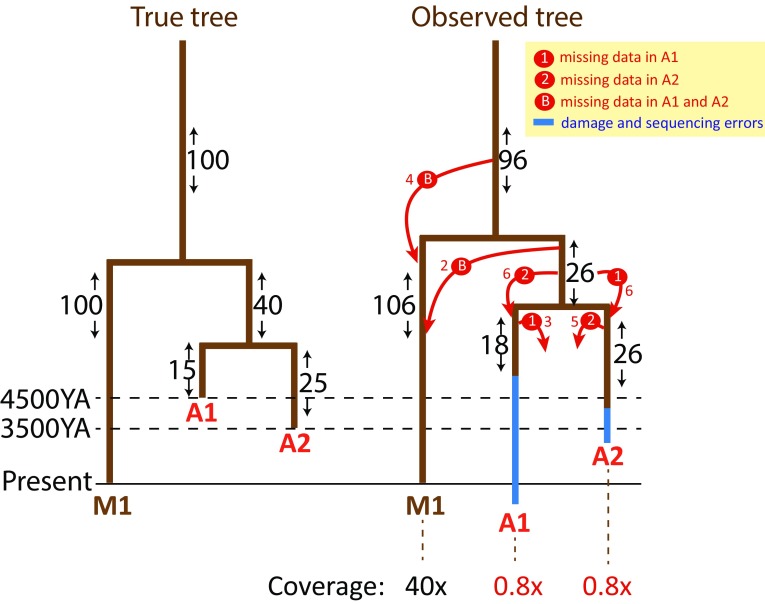
Effect of low coverage of the data and post-mortem damage on the inferences of branch lengths and on the phylogenetic mapping of mutations to the tree. A general example of phylogenetic relationships between one high coverage modern (M1) and two low coverage ancient (A1 and A2) samples is shown. The number of mutations mapping to each branch is shown on both trees. The change in numbers of mutations mismapped due to low coverage on each branch of the tree is explained with the *red arrows* that indicate the directionality of the mismapping. A1 is shown to carry more damage-induced mutations than A2. Modified from Poznik et al. (2016), Supplementary Figure 18

Two different approaches, shotgun and hybridization capture-based sequencing, have been used in recent ancient DNA studies to assess the variation of ancient Y chromosomes. In all genome-scale studies, shotgun sequencing is typically used firstly in the screening phase. In case of high percentage (~>10%) of reads mapping uniquely to the human reference genome combined with low clonality of the reads, further shotgun sequencing of the sample to a higher coverage can be generally considered to be an efficient and relatively cost-effective strategy. However, in cases where the content of human genome mapping reads is low (~1 to 10%), enrichment methods that target specifically human-specific DNA have been considered to be a practical solution for getting improved coverage of SNPs that are considered informative in downstream analyses (Haak et al. 2015; Lazaridis et al. 2016; Mathieson et al. 2015). Having sufficient coverage of the Y chromosome SNP data in ancient samples is important for mapping them correctly to the phylogenetic tree and for examining the relationship among multiple ancient samples in parallel. First, low coverage of data can have an undesirable effect on the inferences of the positioning of ancient samples in a tree in relation to high coverage modern Y chromosome sequence data, and, in particular, for the estimation of branch lengths of the phylogeny as exemplified in Fig. 1. Besides the coverage (a measure of how many sites of the reference genome are covered by data in a given sample), it is also the sequencing depth (the number of reads covering a site) at each given site that contributes to the branch length estimates as sites covered by only one or two reads tend to have a high rate of false positive sequencing errors. Second, when pooling from many ancient samples, data analysis methods that rely on allele frequency comparisons of individual SNPs require reasonable number of overlapping sites covered by data in multiple samples. Predictably, the more low coverage samples are included in the analyses the less overlapping SNPs remain available for downstream data analyses.

A number of Y chromosome sequences covered in this review have been generated with shotgun sequencing approach. These include the four oldest genomes of individuals dated to late Pleistocene as well as a number of remains from Europe and Americas dated to the Holocene period. A substantial proportion of the European and Middle Eastern Neolithic and Bronze Age remains have been sequenced, however, with hybridization-based capture technique. Capture-based methods focus on specific targets in the genome with the aim to raise their relative coverage in the resulting data. The human origins (HO) genome-wide SNP array (Patterson et al. 2012) has been used to genotype more than 600 K SNPs in more than two thousand living individuals from 203 populations (Lazaridis et al. 2014). A subset of 390 K SNPs from this array, including 2258 Y chromosome markers, were targeted later (Haak et al. 2015) in a hybridization-based capture design to assess variation in 69 Europeans living 8–3 thousand years ago. The extended panel of the HO hybridization design now targets 1240 K SNPs genome-wide including 32,681 from the Y chromosome (Mathieson et al. 2015). This approach has been used to assess at a high molecular resolution the phylogenetic affiliation of a large number of 110 ancient Y chromosomes from Europe and Near East (Lazaridis et al. 2016).

**Fig. 2:**
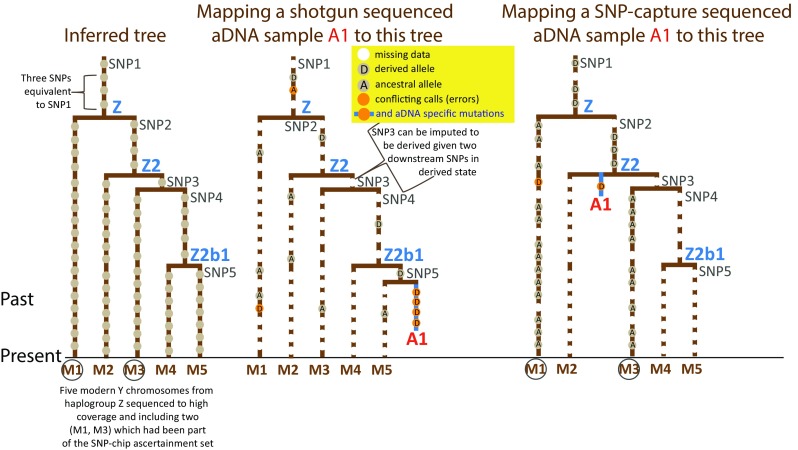
Phylogenetic mapping of a low-coverage ancient DNA sequence to a tree drawn from a general example of a data set of high coverage sequences of modern samples. *Left panel* shows the tree inferred from modern samples. *Middle section* shows the mapping of a sample sequenced by shotgun approach. The *right panel* shows the mapping of the same sample to the tree following SNP-targeted capture approach where modern samples 1 and 3 had been used in the capture design

Several aspects of the capture-based data need to be considered when interpreting the phylogenetic reconstructions based on genotype rather than sequence data. First, the advantage of the capture approach, as mentioned above, is that it allows for the generation of data with higher coverage and higher overlaps among large number of individuals for a given set of SNPs (Pickrell and Reich 2014). This means that even in case of poorly preserved samples, robust haplogroup inferences, supported by multiple phylogenetically equivalent SNPs, can be made. The generic limitation of the approaches that focus on the enrichment-targeted SNPs is the ascertainment bias towards previously discovered variants and that the capture approach does not enable the user to discover novel variants and clades that have either become extinct or that have been not presented in the ascertainment set used for the design of the panel of SNPs to be captured (Fig. 2). Although the 32,681 Y-SNPs of the HO-1240K design include a large number of Simons Genome Diversity Project (Mallick et al. 2016) and ISOGG (International Society of Genetic Genealogy, http://isogg.org/tree/) catalogued variants these would entail, by design, predominantly annotations of the clades that are common in present day populations.

Because most personal genomes that have been annotated by ISOGG come from individuals of European or North American descent the capture enriched for ISOGG SNPs is best suited for the study of European Y chromosome diversity while being less efficient for the study of other regions. But also in Europe it should be noted that clades that have become infrequent or extinct over time due to extensive admixture or population replacement would have less chance to be recognised with the SNP-targeting capture approach. If the parental clade of an extinct branch has extant sister-clades that have been characterised in the annotated databases the aY sequence from that extinct clade will be characterised at the level of the parental clade (Fig. 2). While the most common Y chromosome haplogroups in Europe are represented in the HO-1240K SNP array by many phylogenetically equivalent SNPs they can be robustly recognised and called even in samples with large number of missing data, the haplogroups of other continental regions are less completely characterised in SNP arrays and therefore shotgun approach may be preferable for the ancient DNA (aDNA) study of Y chromosome variation in those areas. As the accessible regions of the Y chromosome that are commonly used in genetic studies are haploid and do not undergo recombination imputation is more efficient in Y chromosome branches represented by multiple equivalent SNPs than it is in autosomal loci and therefore shotgun sequence data with fairly low coverage can be used for unbiased assessment of phylogenetic affinities of aY lineages. However, when using imputation, particularly in the more terminal branches of the tree, one should be cautioned that some of the ancient samples may in fact derive from clades that share only part of the SNPs defining the extant branch of the Y tree and not all of the SNPs.

Another complicating factor for determining Y chromosome haplogroups from ancient DNA data is that it can be challenging to distinguish true mutations from those induced by damage, particularly in case of C to T and G to A substitutions (Gilbert et al. 2003; Hofreiter et al. 2001). A commonly used strategy for dealing with post-mortem damage in ancient DNA studies is the removal of transitions which are the main targets of deamination-induced miscalls via uracil DNA glycosylase treatment or data filtering (Orlando et al. 2015). As transitions occur naturally more frequently than transversions their total removal can lead, however, to dramatic losses of data and thereby loss of phylogenetic resolution, particularly in cases where the sub-clade-defining branches are short. Therefore, aY sequences with low coverage (<0.1×) may not contain enough informative transversions to allow for robust haplogroup assignment at a resolution that would be useful for testing cases of genetic continuity or admixture. Capture approach may yield better coverage at targeted sites but it should be also cautioned that some SNP-targeting capture designs can be enriched for transitions due to the removal of strand ambiguous G<->C and A<->T transversions from the design of SNP-chips.

High coverage Y chromosome sequences, whether from modern or ancient samples, can be used to draw trees with informative branch lengths. With low coverage data generated from ancient human remains, it is typically not possible to make a clear distinction between damage-affected sites and true mutations (Fig. 1). This ambiguity makes it either highly problematic or impossible for the user to determine the lengths of the private branches of the ancient samples. Capture-based methods that are designed to target sequences surrounding a restricted number of known SNPs have a number of advantages, as reviewed earlier (Pickrell and Reich 2014), while at the same time being limited to detect only variants that are included in the capture design. This means that these methods do not allow for the discovery of new variants nor to determine the length of the private branches of the Y chromosome tree. The capture designs targeting large regions of DNA that uniquely map to the Y chromosome, such as the *BigY* design of FamilyTree (https://www.familytreedna.com/learn/wp-content/uploads/2014/08/BIG_Y_WhitePager.pdf) which has 67,000 probes that enable the generation of 4.36 Mbp sequence data from “globally covered” regions of the Y chromosome, or the smaller scale capture design of 500 kb non-recombining regions of Lippold et al. (2014), have been successfully applied on modern samples. These capture approaches are able to detect new, previously uncharacterized variants within the captured regions but they are yet to be shown to work efficiently on ancient DNA. Hybridization enrichment approach targeting chromosome 21 has been successfully applied on the ~40 thousand year-old (KYA) human remains from Tianyuan Cave outside Beijing, China (Fu et al. 2013) suggesting that similar approaches can be applied in the future also on accessible (non-ampliconic) regions of the Y chromosome.

Although HTS methods enable us to generate sequence data from short ancient DNA molecules downstream analysis of extremely short Y chromosome mapping reads is limited by the complexity of the Y chromosome which includes large number of repeats and regions that map to other chromosomal regions. This means that female individuals may yield calls for Y chromosome SNPs surrounded by sequence highly homologous to the X chromosome. Inclusion of SNPs that map outside unique single-copy regions of the Y chromosome confounds phylogenetic mapping of ancient DNA samples and may also bias the analyses based on branch lengths and quantitative estimates of sequence divergence between samples. The approach most typically used in HTS studies of Y chromosome diversity is to filter out SNPs from high homology regions, typically converging to X chromosome degenerate regions of a total size <10 Mbp (Francalacci et al. 2015; Karmin et al. 2015; Poznik et al. 2013; Wei et al. 2013). Several attempts to infer Y chromosome mutation rate from these uniquely mapping regions using ancient DNA data have been made, as reviewed in the paper by Oleg Balanovsky in this volume.

## Genetic history of Eurasian Y chromosomes

**Fig. 3:**
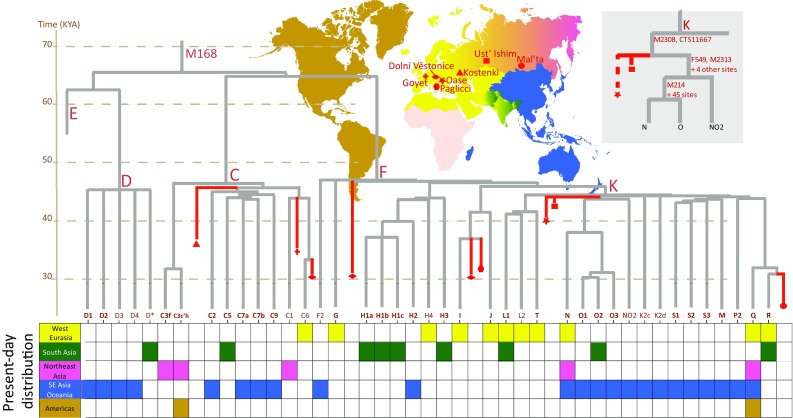
Human Y chromosome diversity outside Africa 20–50 thousand years ago. The branching structure of 41 extant Y chromosome clades inferred to be older than 30,000 years is shown according to the tree based on high coverage Y chromosome sequences presented in Karmin et al. (2015), Figure S9. The phylogenetic mapping to this tree of nine late Pleistocene ancient Y chromosomes that predate the Last Glacial Maximum—from Ust’Ishim (Fu et al. 2014), Oase (Fu et al. 2015), Kostenki (Seguin-Orlando et al. 2014), Malt’a (Raghavan et al. 2014b), Dolní Věstonice, Goyet, and Paglicci (Fu et al. 2016) sites—is shown in red. The zoomed in version of the sub-tree relating Oase, Ust’Ishim with extant variation of the N, O and NO2 clades is shown with branch-defining marker names [modified from (Poznik et al. 2016)] next to the geogrpaphic map displaying the locations of the four ancient sites. The *colour-coded* distribution of Y chromosome haplogroups in present day populations ignores cases of recent admixture. Haplogroup names here and in other figures follow the nomenclature suggested in Karmin et al. (2015)

Consistent with generally higher genetic diversity in African populations, the highest number of deeply splitting branches of human Y chromosome tree can be found in African populations (Cruciani et al. 2011; Mendez et al. 2013; Poznik et al. 2013; Wei et al. 2013). The extant variation in other continents is mainly restricted to three branches of the M168 clade: D, C and F (Hallast et al. 2015; Karmin et al. 2015; Poznik et al. 2013, 2016; Scozzari et al. 2014; Wei et al. 2013). Genetic variation within each of these three clades coalesces to a single founding lineage within 40–60 KYA time depth, consistent with the Out of Africa (OOA) dispersal model (Stringer 2002). In addition to these three clades, the populations of Near East and Europe also show the presence of young sub-clades of haplogroup E which are likely to reflect recent episodic gene flow from Africa within the last 25 KYA and a range expansion of a sub-clade E2a1-V13 in Europe within the last few thousand years (Cruciani et al. 2007). The three basal Eurasian branches D, C and F split further into ~40 extant branches that are older than 30KYA (Fig. 3). Knowing the age of the ancient human remains whose genomes have been sequenced, we can map their Y chromosomes on the phylogenetic tree relating these extant branches and ask whether they are consistent with the inferences of ancestral variation made from modern data or whether they represent distantly related clades that have left no living descendants. In Fig. 3, aY sequences with known radiocarbon dates are mapped, on the basis of the derived alleles they share with modern sequences, on a tree summarizing variation in modern whole Y chromosome sequence data (Scozzari et al. 2014; Karmin et al. 2015; Hallast et al. 2015; Batini et al. 2015; Poznik et al. 2016). The nine oldest ancient Y chromosomes that have been sequenced so far at sufficiently high coverage to allow for phylogenetic mapping are from northern parts of Eurasia (Fu et al. 2014, 2015, 2016; Raghavan et al. 2014b; Seguin-Orlando et al. 2014). Notably, all nine of these aY sequences can be assigned to one of the three major founding lineages that have been inferred from the analyses of extant Y variation. This good match between the ancestral lineages that are observed and those expected from present-day variation to have existed in the time frame of 20–50 thousand years ago supports the view of a robust recovery of non-Africans from extremely low values of male effective population size during the OOA bottleneck (Lippold et al. 2014).

Two of the oldest human Y chromosomes sequenced so far, Ust’Ishim Man (Fu et al. 2014) and the Oase Man (Fu et al. 2015), are both placed near the root of haplogroup K, a sub-clade of F, which is globally the most frequent Y chromosome lineage alive today. K is an ancestral group that unites a number of regional haplogroups that are found widely spread today in Europe, East Asia, Oceania, and Americas. Notably, however, the Y chromosomes of these two ancient Eurasian colonists are not exactly equidistant to all living descendants of haplogroup K that are found today in the world. Both Ust’Ishim and Oase men share the derived allele of a marker M2308 (Poznik et al. 2016) which defines the basal root of two common haplogroups N and O (Fig. 3). These M2308-derived haplogroups have a wide spread today in Eurasia, extending from Finno-Ugric populations of Northeast Europe to Tibeto-Burman, Austroasiatic and Austronesian speaking groups of South and East Asia (Ilumae et al. 2016; Poznik et al. 2016). Although the Ust’Ishim and Oase men were separated from each other about 5 thousand years in time and about 5 thousand kilometres in space these two sampling points we have from the earliest period of peopling of Eurasia suggest some level of continuity in certain Y chromosome lineages that exist in present-day populations of Eurasia. In contrast, the analyses of autosomal genomes have shown that both Ust’Ishim and Oase men were equidistant to East and West Eurasian populations and therefore unlikely to have contributed substantially to later humans in geographic regions where they were living in (Fu et al. 2014, 2015). The phylogenetic affiliation of these early Eurasian men with an NO-related clade is notable because phylogeographic inferences made from present day Y chromosome variation have highlighted other haplogroups, I and R1-M173, as signatures of the genetic legacy of Palaeolithic humans in West Eurasia (Semino et al. 2000) while the origin of haplogroups N and O (Rootsi et al. 2007), as well as other sub-clades of haplogroup K (Karafet et al. 2015), was deemed most likely to be, on the grounds of the highest genetic diversity, in Southeast Asia.

**Fig. 4:**
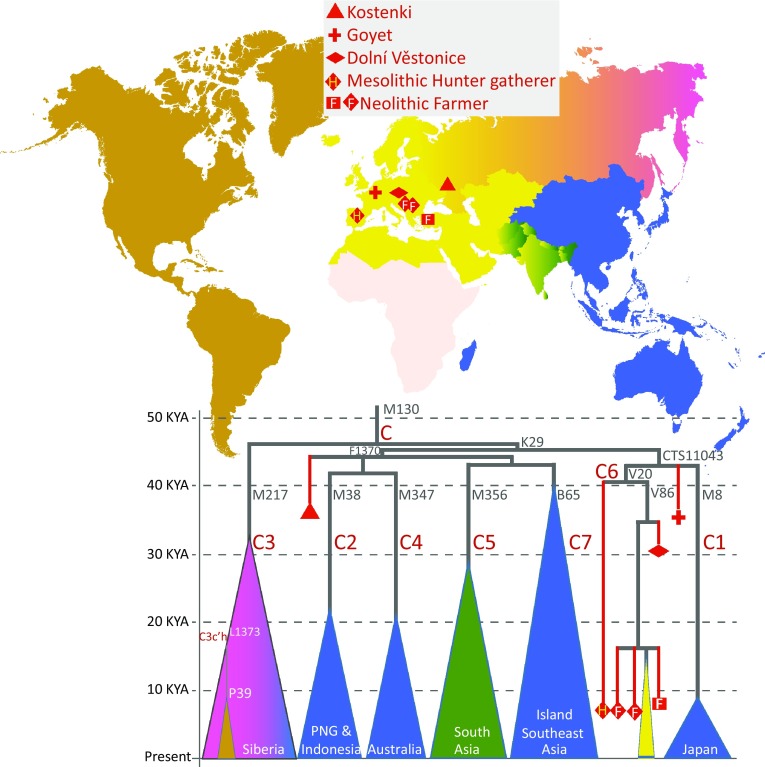
Major sub-clades of Y chromosome haplogroup C in ancient and present-day populations. The structure of the major sub-clades is drawn in proportion to their coalescent time (*the tip of each triangle*) estimated from high coverage genomes of present-day populations (Bergstrom et al. 2016; Karmin et al. 2015; Poznik et al. 2016; Scozzari et al. 2012). The phylogenetic mapping of ancient Y chromosomes (Gamba et al. 2014; Mathieson et al. 2015; Olalde et al. 2014; Seguin-Orlando et al. 2014) is shown with *red symbols*. Haplogroup names are shown in *brown font* and haplogroup-defining SNP-marker names in *grey font* next to relevant branches. The key areas of the present-day spread of the haplogroups are indicated with *colour* and *white text inside the triangles*. *PNG* Papua New Guinea

The 37 KYA Kostenki-14 Man, found on the western bank of river Don near Voronezh in Southwest Russia, and whose genome has been sequenced to an average depth of 2.8×, descended from a population that was more closely related to modern Europeans than to East Eurasians (Seguin-Orlando et al. 2014). Yet, his Y chromosome belonged to haplogroup C which is extremely rare or absent in most European populations sampled today (Semino et al. 2000). So, although his autosomal genes show the highest affinity to European present-day populations his Y chromosome is different. Haplogroup C is common today in populations of Siberia, Southeast Asia and Oceania (Bergstrom et al. 2016; Karafet et al. 2001, 2002; Kayser 2010). This disparity may suggest, in line with similar findings of mitochondrial DNA haplogroup M lineages in pre-Holocene remains from Europe, that peopling of Europe involved several population replacements and turnovers (Posth et al. 2016). Furthermore, the occurrence of haplogroup C lineages different from the Kostenki type, those belonging to a rare C6-V20 (Scozzari et al. 2012) sub-clade (Fig. 4), in Iberian hunter-gatherer sample from La Brana (Olalde et al. 2014) and in three Neolithic farmer samples from Anatolia and Central Europe (Mathieson et al. 2015) shows that a diverse set of haplogroup C lineages may have been common and widely spread throughout Eurasia before Middle Holocene. Overall, this newly emerging aDNA evidence from late Pleistocene shows that the Y chromosome pool of West Eurasia has undergone significant changes over time and that inferences made from present-day genetic variation about the time and place of origin of Y chromosome haplogroups can be imperfect.

**Fig. 5:**
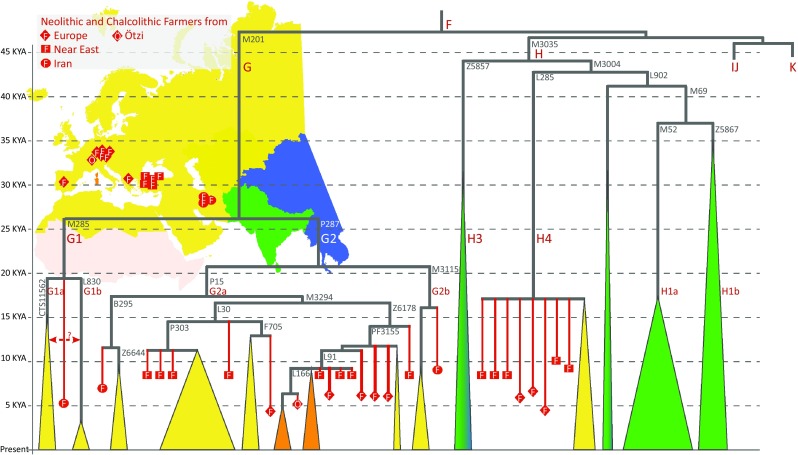
Major sub-clades of Y chromosome haplogroups G and H in ancient and present-day populations. The structure of the major sub-clades is drawn in proportion to time according to estimates from high coverage genomes of present-day populations (Hallast et al. 2015; Karmin et al. 2015; Poznik et al. 2016) http://isogg.org/tree/, https://www.yfull.com/). The phylogenetic mapping of ancient Y chromosomes (Allentoft et al. 2015; Broushaki et al. 2016; Fu et al. 2016; Gunther et al. 2015; Haak et al. 2015; Hofmanova et al. 2016; Lazaridis et al. 2014, 2016; Mathieson et al. 2015) is shown with *red symbols*. Haplogroup-defining marker names are shown in *grey font* next to relevant branches

Besides the haplogroup C lineages that are atypical to present-day populations living in the area the Y chromosome pool of the early Holocene hunter-gatherer and farmer populations of Europe and Middle East was characterised by a diverse set of haplogroups, such as G, H, I, J and R, which are restricted in their present-day distribution by and large to Europe, Middle East, North Africa, South and Central Asia. The geographic patterns of haplogroup G distribution in present-day populations have been suggested to reflect the spread of Anatolian farmers to Europe (Semino et al. 2000). Its sub-clades are most frequently found today in the Caucasus and West Asia while being rare in Europe (Cinnioglu et al. 2004; Firasat et al. 2007; Haber et al. 2012; Nasidze et al. 2008; Rootsi et al. 2012; Yunusbayev et al. 2012). Ancient DNA evidence from Anatolia and Iran confirms that G, along with H, was the most common Y chromosome haplogroup of the early farmers in these areas (Fig. 5) as well as being characteristically frequent in European Early Neolithic populations who also show low autosomal genetic distances with Anatolian farmers (Broushaki et al. 2016; Hofmanova et al. 2016; Lazaridis et al. 2016; Mathieson et al. 2015). Coherent with the tight clustering of the autosomal genome of the Tyrolean Iceman, nicknamed Ötzi, together with present-day populations of Sardinia and Corsica (Keller et al. 2012), and the claims that Sardinians represent the genetic continuity from the early farmers of Europe (Sikora et al. 2014; Skoglund et al. 2012), Ötzi’s Y chromosome lineage, G2a-L166, descends from the G2a-L91 lineage that was common among Anatolian Farmers 8 KYA (Lazaridis et al. 2016). Today, the G2a-L91 sub-clade has survived in Europe as a rare lineage with the highest incidence in Sardinia and Corsica (Francalacci et al. 2013; Keller et al. 2012).

In contrast to haplogroup G, the geographic distribution of haplogroup H is presently almost entirely restricted to South Asia, while one of its sub-clades, H4-L285 (Fig. 5), can be detected as an extremely rare lineage in some European populations. H4-L285 has also been found in the aY sequences of the Anatolian and Levantine farmers as well as in Iberian Chalcolithic samples (Gunther et al. 2015; Lazaridis et al. 2016). Overall, the comparisons of early and middle Holocene versus present-day distributions of haplogroup G and H suggest that as characteristic markers of the early farmer populations of Middle East they were introduced to Europe by the expanding Anatolian farming populations. Their frequency has remained high in some geographically isolated areas such as the Caucasus, Sardinia, Corsica, whereas their frequency in main parts of Europe dropped later due to inflow of other Y chromosome lineages.

**Fig. 6:**
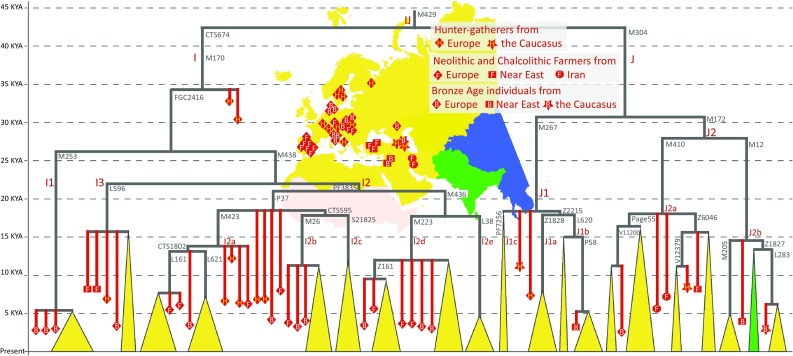
Major sub-clades of Y chromosome haplogroups I and J in ancient and present-day populations. The structure of the major extant sub-clades of haplogroups I and J is shown by *triangles* the tips of which are drawn in proportion to time according to coalescent time estimates from high coverage genomes of present-day populations (Hallast et al. 2015; Karmin et al. 2015; Poznik et al. 2016) http://isogg.org/tree/, https://www.yfull.com/). The *colour of each triangle* reflects the main geographic areas of its spread according to the map shown in the *middle of the plot*. The phylogenetic affiliations of ancient Y chromosomes (Allentoft et al. 2015; Gamba et al. 2014; Gunther et al. 2015; Haak et al. 2015; Hofmanova et al. 2016; Jones et al. 2015; Lazaridis et al. 2016; Mathieson et al. 2015; Skoglund et al. 2012) are shown with *red symbols*. Haplogroup-defining marker names are shown in *grey font* next to relevant branches

Haplogroup I frequency distribution is largely restricted to Europe where two major sub-clades I1 and I2 describe most of the extant variation (Fig. 6). It has been proposed that this restricted geographic area of its spread reflects local continuity since the Palaeolithic hunter-gatherers (Rootsi et al. 2004) standing in contrast to the geographic area of the highest frequency and diversity of its sister-clade J which alongside with the cline of haplogroup G frequency has been interpreted as a reflection of demic diffusion of farmers from Middle East to Europe (Semino et al. 2000). The ancient DNA evidence that has started to emerge recently confirms that haplogroup I was indeed common in Palaeolithic hunter-gatherers of Europe (Fu et al. 2016; Lazaridis et al. 2014); but it also points to a more complex picture with both hunter-gathers and farmers carrying haplogroup I as well as J lineages. The minor sub-clade of haplogroup I, I3-L596 (Fig. 6), has been found spread across a wide geographic area in Early and Middle Holocene samples, being found in Anatolian Farmers (Lazaridis et al. 2016) as well as in Scandinavian hunter-gatherers from Motala (Mathieson et al. 2015). I1-M253 lineages which are common in Scandinavia today, at 25–35% frequency (Rootsi et al. 2004), coalesce to a recent common founder at 5KYA. Similarly to its present day peak frequency area aY sequences falling to this clade are by and large restricted to three Nordic Late Neolithic and Bronze Age samples (Allentoft et al. 2015). Interestingly, one Trans-Danubian Early Neolithic (7.6–6.9KYA), LBK, sample from Hungary has also been recovered with I1-M253 affiliation (Szecsenyi-Nagy et al. 2015), suggesting that I1-M253 lineages may have been brought to Scandinavia by Neolithic farmers rather than representing local continuity of a pre-Holocene pool of Y chromosome lineages. I2-PF3835 lineages were, second only to haplogroup G, among the most common Y chromosome groups in early farmers of Central Europe (Gamba et al. 2014; Mathieson et al. 2015; Szecsenyi-Nagy et al. 2015). Lineages distantly related to the extant sub-clades of haplogroup I2a-M423, which are common in Western (I2a-L161) and Eastern (I2a-L621) Europe today have been found in the aY sequences of hunter-gatherers from Switzerland, Hungary and Scandinavia as well as in Neolithic and Bronze Age samples from Hungary, Germany and Iberia suggesting some level of regional continuity (Haak et al. 2015; Jones et al. 2015; Mathieson et al. 2015).

Haplogroup J, which, on the basis of its present-day clinal frequency distribution has been associated with the early spread of farming to Europe (Rosser et al. 2000; Semino et al. 2000), has not been detected so far in European Neolithic context, instead, this haplogroup is found in hunter-gatherers from geographically distant areas, from the Caucasus and Karelia (Fig. 6), as well as in two early farmers from Iran and one from Anatolia (Jones et al. 2015; Lazaridis et al. 2016; Mathieson et al. 2015). In Central and Western Europe, J lineages start to emerge in the Bronze Age, likely being part of the demographic processes and population movements initiated from the North Caucasus area during that period. It is possible that these recent processes also introduced to Europe sub-clades of haplogroup E (Cinnioglu et al. 2004; Cruciani et al. 2007; Trombetta et al. 2015) which according to recent ancient DNA evidence was a characteristic haplogroup of the Natufians, pre-pottery farmers of Levant and Ethiopians (Gallego Llorente et al. 2015; Lazaridis et al. 2016).

**Fig. 7:**
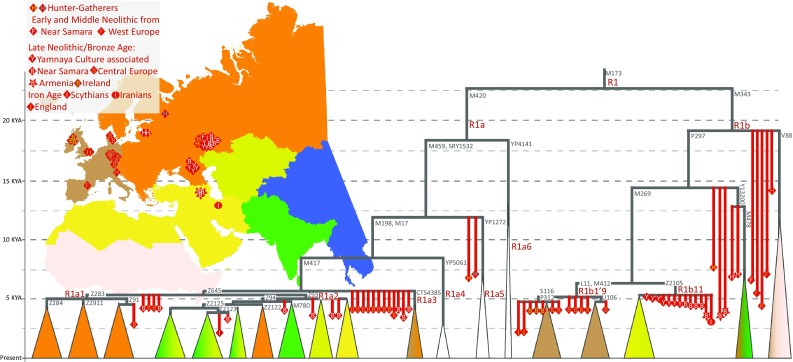
Major sub-clades of Y chromosome haplogroups R1a and R1b in ancient and present-day populations. The structure of the major extant sub-clades of haplogroups R1a and R1b is shown by *triangles* the tips of which are drawn in proportion to time according to coalescent time estimates from high coverage genomes of present-day populations (Batini et al. 2015; Cruciani et al. 2010; Hallast et al. 2015; Karmin et al. 2015; Poznik et al. 2016), http://isogg.org/tree/, https://www.yfull.com/). The *colour of each triangle* reflects the main geographic areas of its spread according to the map shown in the *middle of the plot*. The phylogenetic affiliations of ancient Y chromosomes chromosomes (Allentoft et al. 2015; Broushaki et al. 2016; Cassidy et al. 2016; Haak et al. 2015; Jones et al. 2017; Lazaridis et al. 2016; Mathieson et al. 2015; Schiffels et al. 2016) are shown with *red symbols*. Haplogroup-defining marker names are shown in *grey font* next to relevant branches. Four Yamnaya, one Samara Bronze Age and two Nordic Bronze Age R1b-M269 samples could not be placed on the tree as they lacked coverage at M412, L11, and Z2105 SNPs

Y chromosome haplogroup R1b-M343 is currently the most common haplogroup among present day populations of Western Europe. It has a peak frequency of ~90% in the Basques who have been considered to represent a relict descendant group, by its genes and languages, of a pre-Neolithic population of Europe (Cavalli-Sforza et al. 1994; Richards et al. 1996). The decreasing northwest to southeast frequency gradient of haplogroup R1b-M343 was interpreted first to have resulted from admixture of Mesolithic hunter-gatherers with Neolithic farmers carrying haplogroups J and G (Rosser et al. 2000; Semino et al. 2000). We now know from whole Y chromosome sequencing studies of modern samples that the coalescent time of the most common European sub-clade of R1b-M269 is shallow, 5–7 thousand years (Batini et al. 2015; Hallast et al. 2015; Karmin et al. 2015; Poznik et al. 2016). From the aDNA studies we have learned that the oldest R1b-M343 lineages, including 14 KYA Villabruna Man from Italy (Fu et al. 2016) and three European hunter-gatherers and three early farmer samples (Fig. 7), did not belong to the R1b-M269 sub-clade. According to the ancient DNA evidence, the R1b-M269 lineages did not, in fact, become common in Europe before the Late Neolithic/Bronze Age (Allentoft et al. 2015; Haak et al. 2015; Mathieson et al. 2015). We also know that modern-day Basques have the highest affinity in their autosomal genes to the early farmers of Atapuerca from Spain (Gunther et al. 2015) while their Y chromosomes arguably reflect a more recent male-specific admixture from Eastern Europe, from the areas of the distribution of Yamnaya Culture (Goldberg et al. 2017; Haak et al. 2015).

In contrast to preceding Early and Middle Neolithic sections of time, a large proportion of the Y chromosomes recovered from Bronze Age remains of Central Europe, Northern Caucasus and the Steppe belt of Russia belong to a couple of sub-clades of haplogroups R1a-M420 and R1b-M343 (Fig. 7). Late Neolithic, Early Bronze Age and Iron Age samples from Central and Western Europe have typically the R1b-L11, R1a1-Z283 and R1a-M417 (xZ645) affiliation while the samples from the Yamnaya and Samara neighbourhood are different and belong to sub-clades R1b11-Z2105 and R1a2-Z93 (Allentoft et al. 2015; Cassidy et al. 2016; Haak et al. 2015; Mathieson et al. 2015; Schiffels et al. 2016). The R1b11-Z2015 lineage is today common in the Caucasus and Volga-Uralic region while being virtually absent in Central and Western Europe (Broushaki et al. 2016). Interestingly, the earliest offshoot of extant haplogroup R1b-M343 variation, the V88 sub-clade, which is currently most common in Fulani speaking populations in Africa (Cruciani et al. 2010) has distant relatives in Early Neolithic samples from across wide geographic area from Iberia, Germany to Samara (Fig. 7). In a similar way, early offshoots of the R1b and R1a phylogenies, including R1b lineages derived at P297 and ancestral at M269, and R1a lineages which are derived at M459 while ancestral at M198 and M417 markers have been found in mid-Holocene hunter-gatherer samples in a wide area in Eastern Europe, from Karelia, Latvia and Samara region (Haak et al. 2015; Jones et al. 2017; Mathieson et al. 2015). Extremely rare extant sub-clades of R1a, such as R1a4-YP5061, R1a5-YP1272, and R1a6-YP4141 (Fig. 7), may bear witness to a long-term continuity of such old genetic lineages while the majority of present-day R1a and R1b lineages in West Eurasia derives from just a handful of Late Neolithic/Early Bronze Age male founders.

## Ancient Y chromosomes of the Native Americans

The present-day pool of Native American Y chromosomes is a mixture of haplogroups that derive from pre-Columbian dispersals from Siberia and more recent gene flow from Europe and Africa (Grugni et al. 2015; Kimura et al. 2016; Roewer et al. 2013; Zegura et al. 2004). The diversity derived from the first dispersals is restricted to just two founding lineages within haplogroup Q and one or two in haplogroup C3-M217 (Fig. 3). The lineages specific to Native Americans within these two ancient haplogroups Q and C that have also branches that are commonly found in different parts of Eurasia have been suggested to have reached America by multiple independent dispersal events from Siberia (Lell et al. 2002). Lell et al. considered haplogroup Q, which is the most common clade in both North and South American Native populations, to derive from a migration from ‘Middle’ Siberia where it is highly frequent today. C3-M217, which in Americas is restricted, as a minor haplogroup, to a small number of populations, is the most common Y chromosome haplogroup in Northeast Siberians. Lell et al. (Lell et al. 2002) argued that the different spread patterns of these two haplogroups in both America and in Siberia are the outcome of dual origins of Native Americans implying two early dispersal events with two distinct source populations. Similar levels of STR diversities, however, that have been observed in Native American C3-P39 and Q1a-M3 lineages have been interpreted in favour of both of these haplogroups being part of the initial pool of the first expansion of Native Americans some 10–17 thousand years ago and thus can be viewed as being in line with what is called the single wave model (Zegura et al. 2004). The finding of a rare group of lineages of C3-M217 that share the ancestral allele for the P39 marker in Ecuador has recently reignited the debate about dual origins of Native American haplogroup C and Q lineages (Roewer et al. 2013). The C3-M217 Y chromosomes without the P39 marker were found in association with STR diversity coalescing to 6000 years and Roewer et al. (2013) suggested that their presence could be explained by a secondary wave from East Asia, and possibly, more specifically from Japan, considering that certain similarities in material culture exist between the areas where the C3-M217 lineages are found. However, analyses of autosomal SNPs have not supported the model of additional gene flow from East Asia to Ecuador suggesting that instead the presence of the rare C3-M217 lineages without the P39 marker represents a case of a rare founding lineage that has been lost elsewhere by drift (Mezzavilla et al. 2015).

Ancient DNA studies have cast new light on the debate of Native American origins and shown that the loss of rare lineages in post-contact Native Americans is not unusual and possibly part of the extensive lineage extinction process that has been observed in mitochondrial lineages (Llamas et al. 2016). The analyses of the genome of 24 KYA human remains, recovered from the Mal’ta site near Lake Baikal and shotgun-sequenced to an average depth of 1× (Raghavan et al. 2014b), have shown that Native Americans do have ‘dual ancestry’ but not in the sense of the dual ancestry model of Lell et al. (2002). The autosomal genome of the Mal’ta Boy has close affinity to modern European and Native American populations while being more distant to East Asians which suggests, considering that overall Native Americans are more closely related to East Asians than to Europeans, that the Native Americans derive approximately one-third of their genetic ancestry from a population to which the Mal’ta Boy was related to while two-thirds of their ancestry derives from a different source, closely related to modern East Asian populations. The Y chromosome of the Mal’ta Boy (Fig. 3) is more closely affiliated to West Eurasian R lineages than to East Asian D, C or O lineages. It represents an extinct lineage that derives from the base of haplogroup R closely after the split of the ancestors of haplogroups Q and R. Because most Native American Y chromosomes belong to haplogroup Q, they are more closely related to European Y chromosomes while their maternal lineages (with the exception of rare haplogroup X2a) are all nested within East Asian variation. It should be noted, though, that since both mtDNA and Y are effectively single loci no firm conclusions about the sex-specific nature of the admixture process that lies at the foundation of Native American ancestry can be made from these observations.

**Fig. 8:**
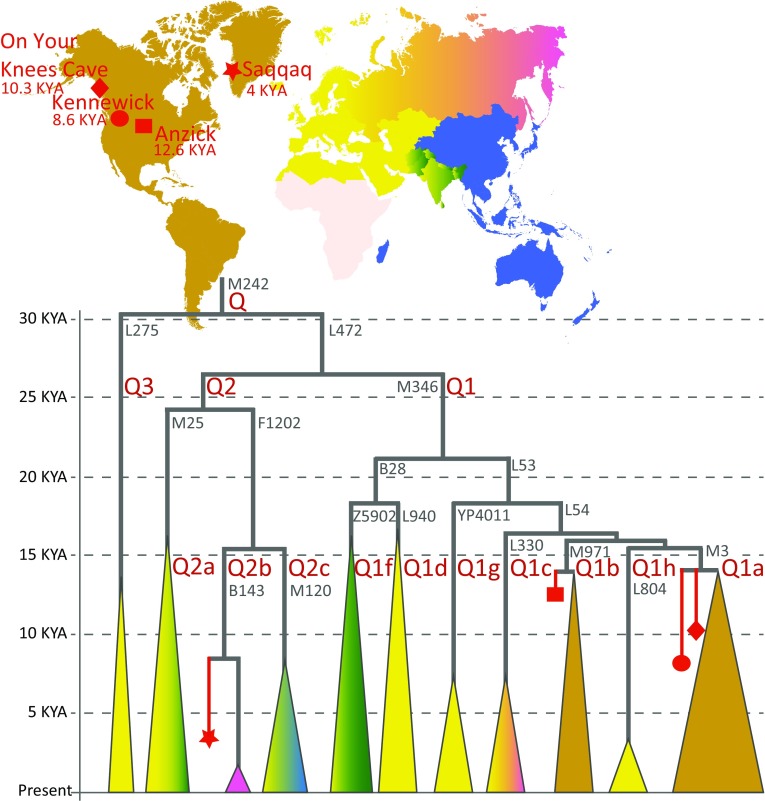
The main branches of Y chromosome haplogroup Q in ancient and present-day populations. The structure of the major sub-clades is drawn in proportion to time according to estimates from high coverage genomes of present-day populations (Karmin et al. 2015; Poznik et al. 2016), http://isogg.org/tree/ISOGG_HapgrpQ.html, https://www.yfull.com/tree/Q/). The phylogenetic mapping of ancient Y chromosomes (Kemp et al. 2007; Rasmussen et al. 2010, 2014, 2015) is shown with *red symbols*. Haplogroup-defining marker names are shown in *grey font* next to relevant branches

Haplogroup Q has two ancient sub-clades, Q1a-M3 and Q1b-M971, which were likely born somewhere in Siberia before the first dispersal into Americas, and which together capture the overwhelming majority of extant Native American Y chromosomes today (Jota et al. 2016; Zegura et al. 2004). Besides these two major clades a number of rare sub-clades of Q that are geographically restricted to Europe, Central and South Asia, or Siberia have been identified (Jota et al. 2016; Karmin et al. 2015; Mallick et al. 2016; Poznik et al. 2016). Targeted PCR-based sequencing of a region surrounding the Q-M242 and Q1a-M3 markers confirmed the affiliation of the 10.3 KYA On Your Knees Cave Man (OYKCM) to haplogroup Q3-L275 (Kemp et al. 2007). Shotgun sequences of two ancient genomes from the Americas, the Anzick Boy (Rasmussen et al. 2014) associated with Clovis Culture, and the Kennewick Man (Rasmussen et al. 2015), have revealed at a finer resolution Y chromosomes that are representative of the present day diversity of haplogroup Q in Americas and together with the OYKCM evidence provide, thus, a direct support to the case that the two extant clades Q1a-M3 and Q1b-M971 have been in the Americas for at least 10,300 years. The Kennewick Man has a Y chromosome that belongs to the most common sub-clade Q1a-M3 while the Anzick’s Y chromosome belongs to the minor Q1b-M971 lineage (Fig. 8).

The third ancient Y chromosome sequence from the Americas, or in fact, technically, from Greenland comes from the Saqqaq site and is dated to 4 KYA. The Saqqaq Man’s mtDNA (Gilbert et al. 2008) and his whole genome, shotgun sequenced to an average depth of 20× (Rasmussen et al. 2010) provided the first direct evidence of a separate Palaeo-Eskimo dispersal event into the Arctic North Americas. The Saqqaq Man’s Y chromosome belongs to Q2b-B143, which is a sub-clade of haplogroup Q that is only distantly related, at time depth >25KYA, to the Q1a-M3 and Q1b-M971 lineages (Fig. 8). The Saqqaq’s Q2b-B143 lineage was not found to be present in a survey of 1863 haplogroup Q lineages from South America making it unlikely to have been among the initial founding pool of Beringian Y chromosomes (Jota et al. 2016). From further ancient DNA analyses, we know that the Palaeo-Eskimos had extremely low genetic diversity, with only a single characteristic mtDNA lineage of haplogroup D2a1 being found in a wide range of sites from Northeast Canada and Greenland dated between 5000 to 700 years before present (Raghavan et al. 2014a). Analyses of autosomal genes and mtDNA of more recent remains associated with the Thule Culture suggest that the Palaeo-Eskimo population was completely replaced by the Neo-Eskimos within less than thousand years ago. Interestingly, though the Saqqaq Man’s Y chromosome lineage may have some continuity in the present day descendants of the Neo-Eskimo dispersal. Greenland Inuits have been shown to carry, at frequencies up to 54% in East Sermersooq, a lineage which is characterised by the NWT01 mutation (Olofsson et al. 2015), a SNP which separates the Y chromosomes of Inuits, who have it, from Athabascans, who do not (Dulik et al. 2012), and which is equivalent to the F1202 SNP that defines the clade that unites the Q2b-B143 and Q2c-M120 sub-clades (Fig. 8). The Y chromosome of the Saqqaq Man has been shown to share a number of SNPs equivalent to B143 with a group of Koryaks from Northeast Siberia (Karmin et al. 2015). It is yet to be revealed, however, whether the Neo-Eskimo Y chromosomes are derived at the SNPs defining the Q2b-B143 branch or whether they represent a yet another sub-clade of Q2-F1202. Further analyses of aY variation across the Americas would help to broaden our understanding of the past dynamics of the male effective population size and show to what extent the lineages present in the past have survived up to the present.

In sum, a number of aDNA studies have already started to reveal the potential of human Y chromosome to inform us about the demographic past complementing the study of autosomal genome and the X chromosome. When compared against the results derived from the analyses of autosomes the unique inheritance patterns of Y, as well as the X chromosome and mtDNA will provide us the opportunity to explore sex-specific dispersal and admixture processes in the future when further sampling will provide larger sample sizes and better coverage of the same geographic areas in time. Further breakthroughs of ancient DNA success in regions like Africa, Southeast Asia and Oceania will be most desirable to tackle broader range of questions about the continuity and nature of sex-specific dispersals and admixture in human evolutionary history. Recent cases of successful retrieval of ancient DNA from Ethiopia (Gallego Llorente et al. 2015), Vanuatu and Tonga (Skoglund et al. 2016) provide some optimism for the retrieval of aY sequence data from warmer climate regions in the future.
